# Ox-Celery

**Published:** 1874-12

**Authors:** 


OX-CELERY. — Our friend, Ditman, the well-known Druggist, under the upper corner of the Astor House, has a new hot drink, which he has named as above. We discovered that it was a very pleasant beef broth, of good strength, and rendered very palatable by the infusion of celery stalks. We look on it as one of the best beverages of the epoch.

